# Mechanism of translation inhibition by type II GNAT toxin AtaT2

**DOI:** 10.1093/nar/gkaa551

**Published:** 2020-06-29

**Authors:** Stepan V Ovchinnikov, Dmitry Bikmetov, Alexei Livenskyi, Marina Serebryakova, Brendan Wilcox, Kyle Mangano, Dmitrii I Shiriaev, Ilya A Osterman, Petr V Sergiev, Sergei Borukhov, Nora Vazquez-Laslop, Alexander S Mankin, Konstantin Severinov, Svetlana Dubiley

**Affiliations:** Centre for Life Sciences, Skolkovo Institute of Science and Technology, Skolkovo 143025, Russia; Center for Precision Genome Editing and Genetic Technologies for Biomedicine, Institute of Gene Biology, Russian Academy of Sciences, 119334 Moscow, Russia; Institute of Gene Biology, Russian Academy of Science, 119334 Moscow, Russia; Institute of Gene Biology, Russian Academy of Science, 119334 Moscow, Russia; Faculty of Bioengineering and Bioinformatics, Lomonosov Moscow State University, Moscow 119992, Russia; Institute of Gene Biology, Russian Academy of Science, 119334 Moscow, Russia; Belozersky Institute of Physico-Chemical Biology, Lomonosov Moscow State University, Moscow 119992, Russia; Centre for Life Sciences, Skolkovo Institute of Science and Technology, Skolkovo 143025, Russia; Center for Biomolecular Sciences, University of Illinois, Chicago, IL 60607, USA; Department of Pharmaceutical Sciences, University of Illinois, Chicago, IL 60607, USA; Department of Chemistry, Lomonosov Moscow State University, Moscow 119992, Russia; Centre for Life Sciences, Skolkovo Institute of Science and Technology, Skolkovo 143025, Russia; Department of Chemistry, Lomonosov Moscow State University, Moscow 119992, Russia; Centre for Life Sciences, Skolkovo Institute of Science and Technology, Skolkovo 143025, Russia; Belozersky Institute of Physico-Chemical Biology, Lomonosov Moscow State University, Moscow 119992, Russia; Department of Cell Biology and Neuroscience, Rowan University School of Osteopathic Medicine, Stratford, NJ 08084-1489, USA; Center for Biomolecular Sciences, University of Illinois, Chicago, IL 60607, USA; Department of Pharmaceutical Sciences, University of Illinois, Chicago, IL 60607, USA; Center for Biomolecular Sciences, University of Illinois, Chicago, IL 60607, USA; Department of Pharmaceutical Sciences, University of Illinois, Chicago, IL 60607, USA; Centre for Life Sciences, Skolkovo Institute of Science and Technology, Skolkovo 143025, Russia; Center for Precision Genome Editing and Genetic Technologies for Biomedicine, Institute of Gene Biology, Russian Academy of Sciences, 119334 Moscow, Russia; Waksman Institute for Microbiology, Piscataway, NJ 08854-8020, USA; Centre for Life Sciences, Skolkovo Institute of Science and Technology, Skolkovo 143025, Russia; Institute of Gene Biology, Russian Academy of Science, 119334 Moscow, Russia

## Abstract

Type II toxin–antitoxins systems are widespread in prokaryotic genomes. Typically, they comprise two proteins, a toxin, and an antitoxin, encoded by adjacent genes and forming a complex in which the enzymatic activity of the toxin is inhibited. Under stress conditions, the antitoxin is degraded liberating the active toxin. Though thousands of various toxin–antitoxins pairs have been predicted bioinformatically, only a handful has been thoroughly characterized. Here, we describe the AtaT2 toxin from a toxin–antitoxin system from *Escherichia coli* O157:H7. We show that AtaT2 is the first GNAT (Gcn5-related N-acetyltransferase) toxin that specifically targets charged glycyl tRNA. *In vivo*, the AtaT2 activity induces ribosome stalling at all four glycyl codons but does not evoke a stringent response. *In vitro*, AtaT2 acetylates the aminoacyl moiety of isoaccepting glycyl tRNAs, thus precluding their participation in translation. Our study broadens the known target specificity of GNAT toxins beyond the earlier described isoleucine and formyl methionine tRNAs, and suggest that various GNAT toxins may have evolved to specificaly target other if not all individual aminoacyl tRNAs.

## INTRODUCTION

Prokaryotic toxin–antitoxin (TA) modules encode a proteinaceous toxin and a cognate antitoxin, which can be a protein or an RNA (1). There is a substantial body of evidence supporting the role of TA modules in plasmid maintenance (2) and abortive phage infection (3). While many studies point towards the importance of TAs in the induction of persistence, virulence, or drug tolerance, the subject remains controversial (4–7).

TAs are classified based on the nature of the antitoxin and mechanism of toxin inactivation. The most diverse type, type II, includes more than a hundred of experimentally verified modules. In addition, thousands of putative type II TA modules have been found bioinformatically in sequenced bacterial and archaeal genomes (8,9). Antitoxins of the type II TA modules are proteins that bind their cognate toxins and form inactive complexes (10). The mechanisms that lead to toxin activation remain elusive and are subject of debate (11). The majority of type II toxins are represented by various RNases that act as translation inhibitors (12). A less common class of type II toxins is comprised of proteins that are characterized by the Gcn5-related N-acetyltransferase (GNAT) fold (8,9). The GNAT class of toxins is monophyletic and contains hundreds of predicted members found in sequenced bacterial genomes (13). The mechanism of action has been identified for five GNAT toxins (13–16). Toxicity of several others was verified *in vivo*, but the targets were not identified (17–21). All biochemically characterized GNAT toxins acetylate the α-amino group of a specific aminoacyl-tRNA and therefore block protein synthesis. Тhe AtaT toxin from *E. coli* O157:H7 targets Met-tRNA^fMet^ (15), ItaT from *E. coli* HS specifically modifies two Ile-tRNAs^Ile^ isoacceptors (13), while three TacT toxins from *Salmonella enterica* Typhimurium exhibit a broader specificity and target various tRNAs (16). Despite significant insights about the activity of these toxins gained in *in vitro* studies, little is known about the effects of GNAT-type toxins on protein translation in the living cell.

The genome of *E. coli* O157:H7 str. Sakai contains two GNAT class toxin–antitoxin modules, *ataRT* (locus tags ECs4308–ECs4307) and *ataRT2* (locus tags ECs4261–ECs4262). While the AtaT toxin of the AtaRT module was thoroughly characterized and shown to modify initiator fMet-tRNA^fMet^ exclusively (15), the functionality of the second module, AtaRT2, was only demonstrated in *in vivo* toxicity tests (21). Phylogenetically, the toxins of AtaRT and AtaRT2 modules are quite distant (29.2% sequence identity) (21) and could be expected to modify different targets inside the cell. In this study, we characterize AtaT2. We show that this toxin specifically acetylates the aminoacyl moiety of Gly-tRNA^Gly^*in vitro* and stalls translating ribosomes at Gly codons in the living cell.

## MATERIALS AND METHODS

### Molecular cloning

Primers used for cloning are listed in Supplementary Table S1.

To construct the plasmids for arabinose-inducible expression of *ataT2* and *ataRT2*, the two genes were PCR-amplified from *E. coli* O157:H7 str. Sakai genomic DNA using AtaT2-F-EcoRI/AtaT2-R-HindIII and AtaR2-F-EcoRI/AtaT2-R-HindIII primer pairs and Phusion DNA polymerase (Thermo Scientific, USA). The PCR fragments were cloned into pBAD33 between EcoRI and HindIII sites resulting in pBAD-*ataT2* and pBAD-*ataRT2*, respectively. The pBAD-*ataT2*-Y139A was constructed by overlap-extension PCR (22) using AtaT2-Y139F-F and AtaT2-Y139F-R mutagenic primers pBAD-*ataT2* as a template. To create pET-str-*AtaRT2*-his plasmid, the *ataRT2* module was PCR-amplified using AtaR2-F-BamHI, and AtaT2-R-XhoI primers, and the resulting PCR fragment was inserted into pET22-strep-his vector (13) between BamHI and XhoI sites. To construct plasmid overexpressing *E. coli* glycyl-tRNA synthetase (GlyRS), the coding region of *glyQ-glyS* was amplified using *E. coli* BW25113 genomic DNA as a template and GlyRS-F-Bam/GlyRS-R-Xho primer pair. The resulting PCR product was cloned into the pET22-str-his vector linearized with BamHI and XhoI restriction enzymes, yielding pET22-str-GlyRS-his.

### *In vivo* toxicity test

*Escherichia coli* BW25113 cells were transformed with pBAD-*ataT2*, pBAD-*ataRT2*, and pBAD*-ataT2*-Y139A vectors and plated on LB agar supplemented with 34 μg/ml chloramphenicol and 1% glucose. A single colony from the freshly transformed plate was inoculated into 10 ml of 2× YT and allowed to grow to OD_600_ ∼ 0.3 at 37°C. Cells were divided into two cultures and grown at 37°C for 30 min in the presence or absence of 1 mM arabinose. Serial 10-fold dilutions of the cultures were plated on LB agar supplemented with 34 μg/ml chloramphenicol and 1% glucose.

### Metabolic labeling assays

Overnight culture of *E. coli* BW25113 carrying pBAD-*ataT2* plasmid or the empty vector pBAD33 were diluted 200-fold in MOPS medium lacking methionine (MOPS(-Met) medium) supplemented with 33 μg/ml chloramphenicol and 1% glucose, and allowed to grow at 37°C to OD_600_ = 0.2. The cells were pelleted, resuspended in fresh MOPS(-Met) medium supplemented with 33 μg/ml chloramphenicol without glucose, and incubated for 30 min at 37 °C with aeration. The culture was then induced with 1 mM arabinose. The samples were collected before induction (0 min) and at 5, 15, 30 and 60 min post-induction for radiolabeling. A 28 μl-aliquots of the cell cultures were combined with 2 μl of MOPS(-Met) medium supplemented with 5 μCi of [^35^S]-methionine. The mixture was incubated at 37°C for 1 min and subsequently spotted onto 35 mm 3MM paper disc (Whatman, USA). For the blank sample, 30 μl of 5% TCA was spotted on a paper disc. The paper discs were boiled for 5 min in 500 ml of 5% TCA. TCA was discarded, and boiling was repeated in fresh 5% TCA. The discs were washed with acetone, air-dried, and placed in scintillation vials filled with 5 ml Ultima Gold scintillation cocktail (Perkin Elmer, USA). Radioactivity was read using a scintillation counter LS 6000 (Beckman Coulter, USA). Background radioactivity was subtracted, and the data were normalized to the empty vector control before the induction (at 0 min).

Overnight cultures of the wild-type *E. coli* BW25113 transformed with pBAD33-*ataT2* or control vector pBAD33, and the mutant *E. coli* strain BW25113 *ΔrelA* harboring pBAD33 were diluted to OD_600_ = 0.1 in MOPS medium containing 0.4% glycerol and 33 μg/ml chloramphenicol and grown at 37°C for 1 h. 50 μl of [^32^P]-orthophosphoric acid (1 mCi/ml, 8500 Ci/mmole, Perkin Elmer, USA) was added to 1 ml of each culture (50 μCi/ml final radioactivity) followed by incubation at 37°C for 40 min. The cultures were induced with 1 mM of arabinose, or serine hydroxamate and incubation continued for 10 min at 37°C. The metabolically radiolabeled nucleotides were extracted and subjected to thin-layer chromatography as described (23), followed by autoradiography.

### Protein expression and purification

An overnight culture of *E. coli* BL21(DE3) cells carrying pET-str-*ataRT2*-his plasmid was diluted 1:200 in 2× YT medium supplemented with 100 μg/ml ampicillin and grown at 37°C with aeration to OD_600_ = 0.8. Protein expression was induced with 1 mM isopropyl β-d-1-thiogalactopyranoside (IPTG). After induction, the culture was maintained at 18°C for 16 h. The cells were pelleted by centrifugation and washed with buffer W (20 mM Tris–HCl pH 8.0, 150 mM NaCl, 1% β-mercaptoethanol).

For purification of the active AtaT2 toxin, the cells were resuspended in the buffer D (20 mM Tris–HCl pH 8.0, 150 mM NaCl, 5 M guanidinium hydrochloride, 1% β-mercaptoethanol) and lysed by sonication. The lysate was then clarified by centrifugation and combined with TALON CellThru Co^2+^-chelating resin (Takara-Clontech). Upon incubation with gentle mixing at 4°C for 4 h, the resin was washed with buffer W. AtaT2 was eluted with elution buffer under denaturing conditions (500 mM imidazole, 20 mM Tris–HCl pH 8.0, 150 mM NaCl, 5 M guanidinium hydrochloride, 1% β-mercaptoethanol). Eluted AtaT2 was refolded by dialysis at 4°C for 16 h in 2 l of dialysis buffer (20 mM Tris–HCl pH 8.0, 20 mM NaCl, 5% glycerol, 1 mM DTT). The fraction of AtaT2, which remained soluble after dialysis, was concentrated, flash-frozen in liquid nitrogen, and stored at −80°C.

For tandem affinity purification, the harvested cells were resuspended in buffer W supplemented with 0.1 mM PMSF and lysed by sonication. The lysate was clarified by centrifugation and incubated with TALON Co^2+^-chelating agarose (Takara-Clontech) with agitation at 1°C for 2 h. The resin was washed with buffer W, and the AtaR2-AtaT2 complex was eluted with imidazole elution buffer (500 mM imidazole in buffer W). The eluted fraction was mixed with Strep-Tactin Superflow Plus agarose (Qiagen, USA) and incubated with agitation at 4°C for 2 h. The resin was washed with buffer W and eluted with desthiobiotin elution buffer (2.5 mM desthiobiotin, in buffer W). Protein fractions were analyzed by Laemmli 12% polyacrylamide gel electrophoresis (SDS-PAGE).

Size-exclusion chromatography of the AtaRT2 complex was performed on a Superdex 200-5/150 GL column (GE Healthcare, USA). A 25 μl-aliquot of the protein fraction eluted from Strep-Tactin resin was applied on the column pre-equilibrated with 10 mM Tris–HCl pH 8.0, 150 mM NaCl buffer. To calculate the molecular weight of the AtaR2–AtaT2 complex, the Gel Filtration LMW and HMW Calibration kits (GE Healthcare, USA) were used. Proteins were eluted at 0.2 ml/min flow rate. Protein fractions were analyzed by 12% SDS-PAGE.

*Escherichia coli* BL21(DE3) cells transformed with pET-str-GlyRS-his and *E. coli* JW1195 strain from ASKA collection (24) were used for purification of GlyRS and Pth, respectively, as described earlier (13).

### *In vitro* translation and toeprinting assays

The DNA template for *in vitro* transcription-translation of *ataR2* was prepared by PCR using the pBAD-*ataRT2* vector template and T7-ataR2-F and T7-ataR2-R primers. The *in vitro* coupled transcription–translation reactions were carried out by PURExpress kit (NEB, USA) using DNA templates encoding *ataR2* or the dihydrofolate reductase (DHFR) (NEB, USA). The *in vitro* translation of firefly luciferase was carried our with *in vitro* transcribed mRNA (Luciferase T7 Control DNA Promega, USA). Before template addition, translation reactions were incubated for 10 min at 37°C in the presence of 0.2 mM of acetyl coenzyme A, with and without 2 μM AtaT2. DHFR- and *ataR2-* translation reaction mixtures were supplemented with FluoroTect™ GreenLys *in vitro* Translation Labeling System (Promega, USA). The fluorescent products of *in vitro* translation reactions were analyzed by 12% SDS-PAGE, and visualized by Typhoon fluorescence gel imager (GE, USA). The enzymatic activity of *in vitro* synthesized luciferase was measured by detection of chemiluminescence in the presence of 0.1 mM d-luciferin with VictorX5 multireader (Perkin-Elmer, USA).

The toeprinting assay was carried out using Rst1 and Rst2 mRNA as templates as described in (25), except that reactions were preincubated for 10 min with 0.4 mM Ac-CoA with or without the addition of 2 μM AtaT2.

### *In vitro* tRNA modification and RNaseT1 treatment

The tRNA aminoacylation was performed in 20 μl of reaction buffer (9 mM Mg-acetate, 5 mM KH_2_PO_4_ (pH 7.3), 95 mM K-glutamate, 5 mM NH_4_Cl, 0.5 mM CaCl_2_, 1 mM spermidine, 8 mM putrescine, 1 mM DTT) supplemented with 5 mM ATP, 0.5 mM of glycine, 1 μM glycyl-tRNA synthetase and 3 μg of the glyV tRNA (Subriden RNA, Rollingbay, WA, USA). The reaction mixture was incubated at 37°C for 30 min, quenched with 20 mM EDTA, and then supplemented with 0.4 mM AcCoA and 2 μM AtaT followed by incubation at 37°C for 10 min. Finally, the reaction was mixed with 1 μM of Pth and incubated at 37° C for additional 10 min. At each step, a 3 μl-aliquot was collected and treated with 1U of RNase T1 (NEB, USA) for 10 min and analyzed by mass-spectrometry using UltrafleXetreme MALDI-TOF/TOF (Bruker Daltonik, Germany) equipped with Nd laser. The MH+ molecular ions of RNase T1-treated tRNA were measured in linear mode; the accuracy of average mass peak measurement was within 1 Da.

### Preparation of the ribosome footprint fragment library

*Escherichia coli* BW25113 cells transformed with pBAD33-*ataT2* or empty pBAD vector were grown overnight in 10 ml of LB medium supplemented with 34 μg/ml chloramphenicol and 1% glucose. The overnight cultures were diluted 200-fold into 200 ml of MOPS medium supplemented with 33 μg/ml chloramphenicol and 1% glucose and were grown at 37°C with vigorous shaking to an OD_600_ of 0.2. The cells were pelleted and resuspended in fresh MOPS medium supplemented with 33 μg/ml chloramphenicol without glucose and grown at 37°C for 30 min, followed by 15 min induction with 1 mM arabinose. The cell cultures were diluted with MOPS medium to OD_600_ = 0.6, cells were harvested by rapid filtration, flash-frozen in liquid nitrogen and cryo-lyzed in 650 μl of lysis buffer (20 mM Tris–HCl pH 8.0, 10 mM MgCl_2_, 100 mM NH_4_Cl, 5 mM CaCl_2_, 0.4% Triton X-100, 0.1% NP-40) containing 65 U of RNase-free DNase I (Roche), 208 U of SUPERase•In™ RNase inhibitor (Invitrogen), and 3 μM GMPPNP (Sigma-Aldrich), as described in (26). The cellular lysates were clarified by centrifugation at 20 000 ×*g* at 4°C for 10 min. The lysates were diluted with the lysis buffer to adjust the OD_260_ to 0.1 in the final volume of 220 μl and treated with ∼450 U of MNase (Roche) at 25°C for 1 h. The reaction was quenched by the addition of EGTA to the final concertation of 5 mM. The monosomal material was purified by sucrose gradient centrifugation. RNA purification and double-stranded DNA deep sequencing library preparation were performed as described in (26).

### Data analysis

The Ribo-seq data were analyzed using the pipeline described by Mohammad *et al.* (27) (available at github.com/greenlabjhmi/2018_Bacterial_Pipeline_riboseq) and custom python/R scripts. The raw reads were trimmed and quality filtered with Skewer 0.2.2 (28). Reads shorter than 15 nt and longer than 40 nt were excluded. We first aligned reads to tRNA or rRNA sequences to filter them out. The remaining reads were uniquely mapped to the MG1655 genome (NC_000913.2). All mapping steps were performed using Bowtie 1.2.2, allowing no more than two mismatches (29). Ribosome density was calculated as the number of 3′-ends of footprints normalized by the total number of uniquely mapped reads (in millions). ORFs shorter than 170 nt and those with the average ribosome density per codon less than 1 (0.33 per nt) were excluded from further analysis. The first 50 nt from the start codon and the last 20 nt before the stop codon of each ORF were also excluded from the analysis (27).

To analyze ribosome distribution over the ORFs lengths, we binned each ORF into 10 segments, and for each segment, obtained percentages of ribosome density along the ORF. 1891 ORFs satisfying the previously mentioned criteria in both toxin and control samples were selected for this analysis. Since the data represent fractions, we treated them as compositions. Thus, the statistical inference was performed after the isometric log-ratio (ilr) transformation. The vectors were compared using paired Hotelling's *T*^2^ test (*P*-value < 0.01). Normality was tested using acompNormalGOF function of the ‘compositions’ R package. For graphical representation, geometric means were used since it is a standard measure of central tendency for compositional data.

The pause score for each nt position was calculated by dividing the ribosome density by the average density on the gene. The positions of the A site of ribosomes were assigned using 11 nt shift from the 3′ ends of footprints. To accurately determine the shift value, we analyzed the average ribosome density for all ORFs aligned at either the start or the stop codons (Supplementary Figure S1). Pause scores for codons in the A site were calculated by averaging the pause score values for all three nucleotides.

## RESULTS

**Figure 1.**
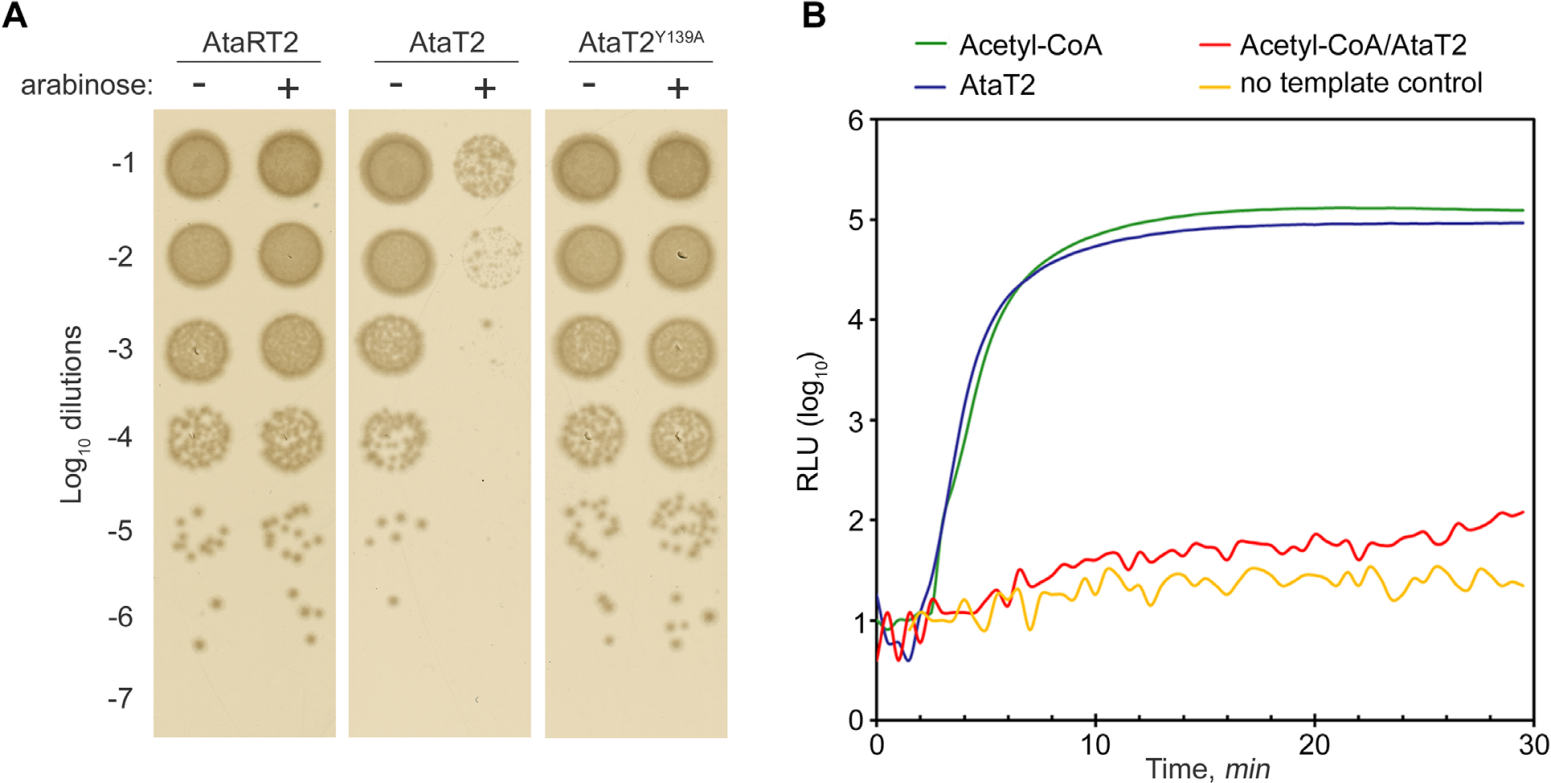
AtaT2 inhibits protein synthesis in an acetyl-CoA-dependent manner. (**A**) Substitution of a conservative acetyl-CoA-binding Y139 abolishes the toxicity of AtaT2 GNAT protein. Serial dilutions of the *E. coli* BW25113 cell harboring pBAD plasmid encoding wild-type or mutant AtaT2 toxin grown for 60 min in the presence or absence of the inducer. Cells expressing *ataRT2* operon were used as a control. (**B**) The activity of firefly luciferase (expressed in relative luminescene units, RLU) synthesized in a coupled *in vitro* transcription-translation system in the absence or presence of AtaT2, acetyl-CoA or both. The time-course of each reaction is shown as colored trace (see legend above the plot).

To test whether the toxicity of AtaT2 depends on its acetyltransferase activity, we expressed the wild-type AtaT2 alone or together with its cognate antitoxin AtaR2, and the catalytically-inactive AtaT2 mutant using *E. coli* BW25113 cells which lack an AtaRT2 module of their own. Genes of the AtaT2 module were introduced on a pBAD plasmid under the control of an arabinose-inducible promoter. In the AtaT2 mutant, the conserved tyrosine Y139, which serves as the general acid during acetyl group transfer of other characterized GNAT toxins (30), was replaced with alanine. While even a transient, 30-minute, expression of wild-type *ataT2* dramatically decreased the number of viable cells in the population, expression of the AtaT2-Y139A mutant, or the toxin–antitoxin pair (AtaRT2) had no impact on cell viability (Figure 1A).

To test whether the two components of the AtaRT2 module form a complex, we tagged the AtaT2 toxin with a strep-tag, and the AtaR2 antitoxin with a hexahistidine tag and the dual construct was expressed in *E. coli* cells. After subjecting the induced cells’ lysate to tandem affinity chromatography, both AtaT2 and AtaR2 were recovered in the eluate (Supplementary Figure S2A). Analysis of the eluted material by size exclusion chromatography revealed a single peak with an apparent molecular weight of 99 kDa (Supplementary Figure S2B), which corresponds to a heterohexameric complex of AtaT2–AtaR2 where toxin dimer is bound to antitoxin tetramer. The same stoichiometry was observed previously for the GNAT-type TA complexes AtaRT and KacAT (31,32). Overall, these data show that the *ataRT2* locus indeed encodes a *bona fide* type II toxin–antitoxin module of the GNAT toxin group.

All GNAT-type toxins studied to date are translational inhibitors, which act by modifying aminoacylated tRNAs (14,15). To test if AtaT2 also inhibits protein synthesis, we purified AtaT2 (see Materials and Methods) and analyzed its functional activity in an *in vitro* translation system using the firefly luciferase reporter assay. As shown in Figure 1B, the addition of either AtaT2 or acetyl-CoA, a donor of the acetyl group used by GNAT enzymes, alone did not affect the luciferase synthesis. In contrast, when the reaction was supplemented with both AtaT2 and acetyl-CoA, the production of luciferase was abolished (Figure 1B). Thus, similar to the other GNAT-type toxins, AtaT2 readily inhibits protein synthesis and such inhibition likely requires acetylation of a specific target.

**Figure 2.**
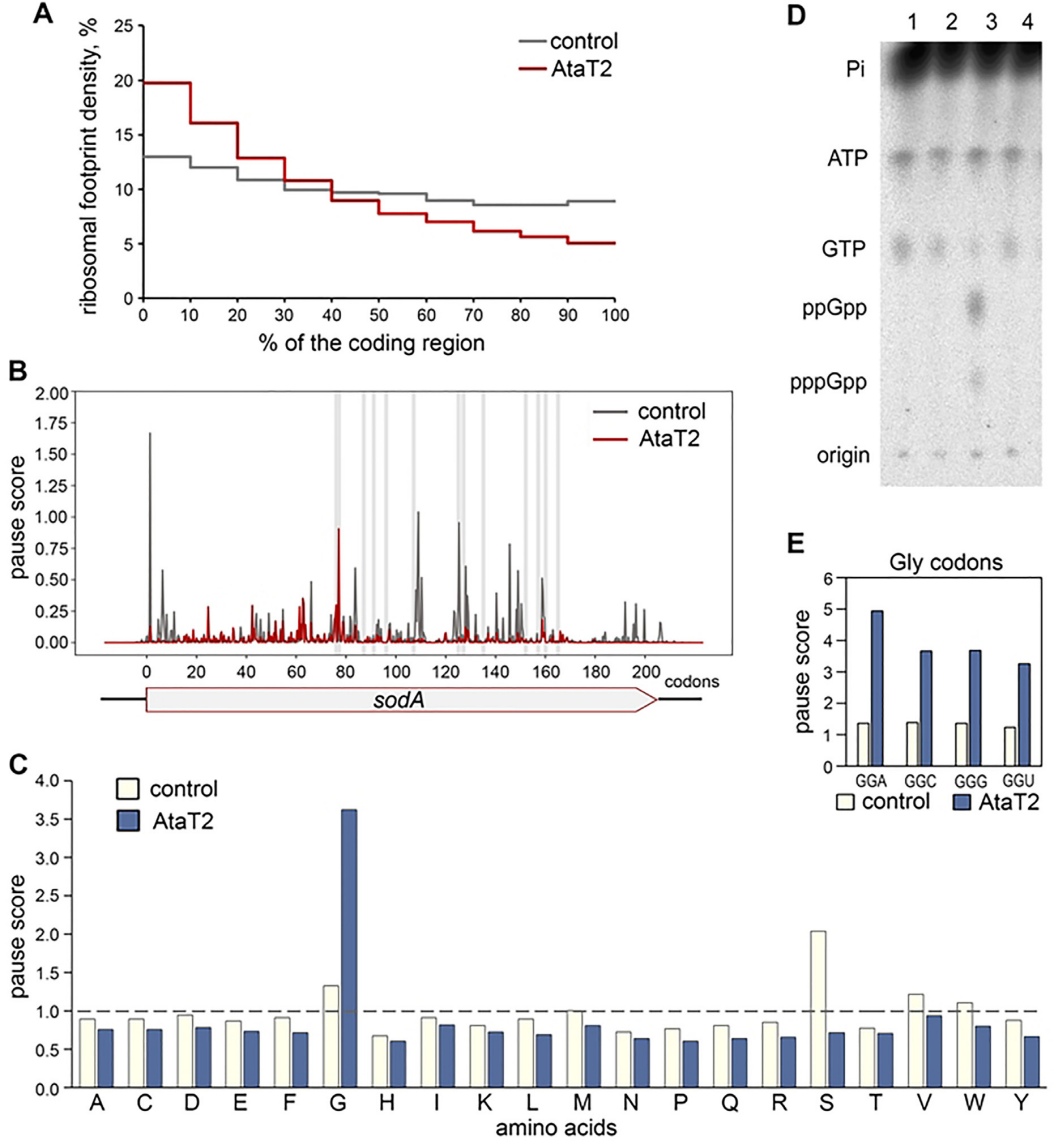
AtaT2 induces ribosomal stalling at Gly codons in the living cell. (**A**) Distribution of ribosome density across coding regions in cells harboring pBAD33-*ataT2* (red) or empty pBAD vector (grey). Each coding region was divided into ten equal segments from 5′ to 3′-end. For every segment percentages of total ribosome density was calculated. The plot represents geometric means. The difference was evaluated with paired Hotelling's *T*^2^ test (*P*-value < 0.01). (**B**) Ribosomal pause score profiles over the *sodA* gene observed in cells expressing AtaT2 (red) and in control (dark grey). Grey vertical lines indicate positions of Gly codons. The pause score values were calculated as ribosome density at each nucleotide of the *sodA* gene divided by the average ribosome density on the gene. (**C**) Average pause scores for specific codons located in the ribosomal A-site. (**D**) AtaT2 expression does not induce ppGpp synthesis. Thin-layer chromatogram of extracts of cells grown in MOPS rich medium supplemented with [^32^P]-orthophosphoric acid and arabinose. Lane 1, *E. coli* BW25113 Δ*relA*/pBAD33; lane 2, *E. coli* BW25113/pBAD33; lane 3, *E. coli* BW25113/pBAD33 in the presence of 1 mM of serine hydroxamate; lane 4, *E. coli* BW25113/pBAD33_*ataT2*. (**E**) Average pause score for individual Gly codons located in the ribosomal A-site.

The mechanisms of cellular toxicity of all type II GNAT toxins have been inferred so far only from the *in vitro* assays. While this approach is informative, it may not adequately reflect the effect of the toxin on protein synthesis in the living cell. To determine how AtaT2 influences translation *in vivo*, we used ribosomal profiling (Ribo-seq), a technique that reveals the distribution of ribosomes on cellular mRNAs with subcodon resolution (33). Previous studies have amply demonstrated the utility of Ribo-seq for elucidating the mechanisms of action of several protein synthesis inhibitors (34–37), including type II toxins (38,39). *E. coli* BW25113 cells were transformed with pBAD-*ataT2* plasmid or an empty pBAD vector. Exponentially growing cells were induced with arabinose for 15 min, a time sufficient to achieve a near-complete inhibition of protein synthesis by AtaT2 (Supplementary Figure S3), then collected by rapid filtration and processed following established Ribo-seq protocols (26,27). The ribosomal footprints were mapped to the genome, and the distribution of ribosomes was compared between toxin-induced and control samples. For the analysis, we selected genes longer than 170 nt and with an average ribosome density higher than 1 per codon. We first analyzed how the ribosome density changes across the length of ORFs. To this end, we partitioned each ORF into 10 segments of equal length (excluding 50 and 20 nucleotides from the 5′- and 3′-end, respectively), and for every segment, calculated percentages of the total number of footprints located within the segment. In the control sample, the ribosomes were distributed relatively evenly across the entire protein-coding region. In contrast, expression of *ataT2* led to a progressive decrease of ribosomal density towards the 3′-end of the coding regions. Only ∼1/3 of the ribosomes that successfully initiated translation were able to reach the end of an ORF in cells synthesizing the toxin (Figure 2A). This result indicates that AtaT2 action leads to inefficient elongation of translation. A similar drop in ribosome density along the ORF length was observed for RNase-type toxins, known to inhibit protein synthesis (38,39).

To investigate whether AtaT2 induces specific changes in distribution of ribosomes across mRNAs, we examined relative ribosome occupancy (pause score) in the 100 ORFs whose mRNAs had the highest average ribosomal density. A comparison between AtaT2 and control samples revealed significant redislocation of ribosomes (exemplified in Figure 2B and Supplementary Figure S4). To analyze systematically the nature of toxin-induced ribosome pausing across the genome, we calculated the mean pause score values for all amino acids in the ribosomal A-site (Figure 2C). In the control sample, a notably higher than average pause score was observed for ribosomes containing Ser codons in the A site (Figure 2С). This type of pausing at ‘hungry’ Ser codons has been previously reported for Ribo-seq experiments in bacteria and most probably is caused by the depletion of charged tRNA^Ser^ cells during cell collection (27,40). In the *ataT2*-expressing cells, ribosomes specifically pause at Gly codons (Figure 2C), thus indicating that translation is arrested when glycine needs to be incorporated in the growing protein chain. No prominent increase in pause score at Ser codons was observed in the AtaT2 sample, probably reflecting the inability of ribosomes to reach the ‘hungry’ Ser codons due to pausing at upstream Gly codons.

The observed ribosomal arrest at a specific codon in the A-site suggests that the cognate tRNA is either uncharged, degraded, or otherwise corrupted and thus can not be used in the translation. Because the accumulation of uncharged tRNA in the cell induces the synthesis of ppGpp (41), we tested whether this alarmone accumulates after induction of *ataT2* expression for 10 min. As expected, we observed ppGpp accumulation in control cells treated with serine hydroxamate, an inhibitor of Ser-tRNA synthetase (Figure 2D). In contrast, ppGpp remained undetectable in cells expressig AtaT2, indicating that the toxin action does not increase the concentration of uncharged tRNA. As direct binding of RelA to uncharged tRNA is required for activation of ppGpp synthesis (42,43), we reasoned that the most likely cause of ribosome pausing at Gly codons observed in our Ribo-seq data is the inability of Gly-tRNA^Gly^ to participate in translation due to specific modification by AtaT2.

Three isoaccepting tRNAs^Gly^ with overlapping specificity are encoded in the *E. coli* genome (44). tRNA^(CCC)^ recognizes the GGG codon, tRNA^(UCC)^ decodes the GGG and GGA codons, while the most abundant tRNA^(GCC)^ is responsible for the recognition of codons GGC and GGU. The pause scores for all glycine codons in the AtaT2 sample were comparable, with slightly stronger pausing at the GGA codon, thus suggesting that AtaT2 can corrupt all aminoacylated Gly isoacceptor tRNAs with some bias toward tRNA^(UCC)^ (Figure 2E).

**Figure 3.**
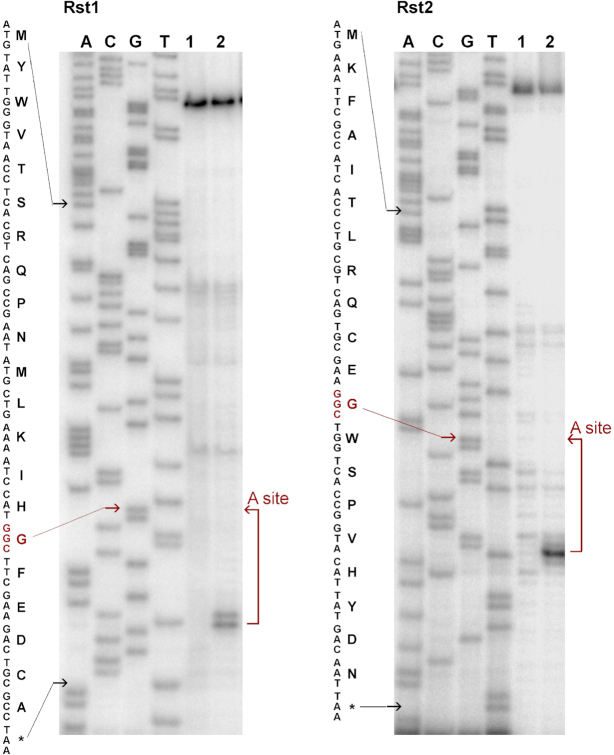
*In vitro* analysis of AtaT2-induced ribosome stalling. Synthetic Rst1 (left panel) and Rst2 (right panel) templates encoding oligopeptides containing all amino acids (25) were translated *in vitro* in the absence (1) or presence (2) of AtaT2 toxin followed by reverse transcription. Samples were separated by sequencing polyacrylamide gel electrophoresis alongside with a sequence ladder and visualized by autoradiography. Specific bands seen in the AtaT2 samples correspond to abortive reverse transcripts accumulated due to the collision of the stalled ribosome and reverse transcriptase. The first nucleotide of the A site-codon is located 13 nt upstream of the position of reverse transcriptase stalling (45). The sequence of peptides encoded in Rst1 and Rst2 templates are shown to the left of the corresponding autoradiogram. The positions of the first nucleotide of the start and stop codons are indicated with black arrows; the position of the first nucleotide of the codon located in the A site of the stalled ribosome is indicated with a red arrow.

We verified the results of Ribo-seq analysis in a cell-free translation system by primer extension inhibition (toeprinting) analysis (45,46). Two synthetic mRNAs encoding different protein products, each containing all 20 amino acids (25), were translated in the presence of acetyl-CoA with or without AtaT2, and the sites of ribosome arrest were detected. In the presence of the toxin, translating ribosomes became arrested specifically at the Gly codon of either one of the two templates (Figure 3), consistent with the scenario that AtaT2-modified Gly-tRNA^Gly^ is incapable of participating in translation.

**Figure 4.**
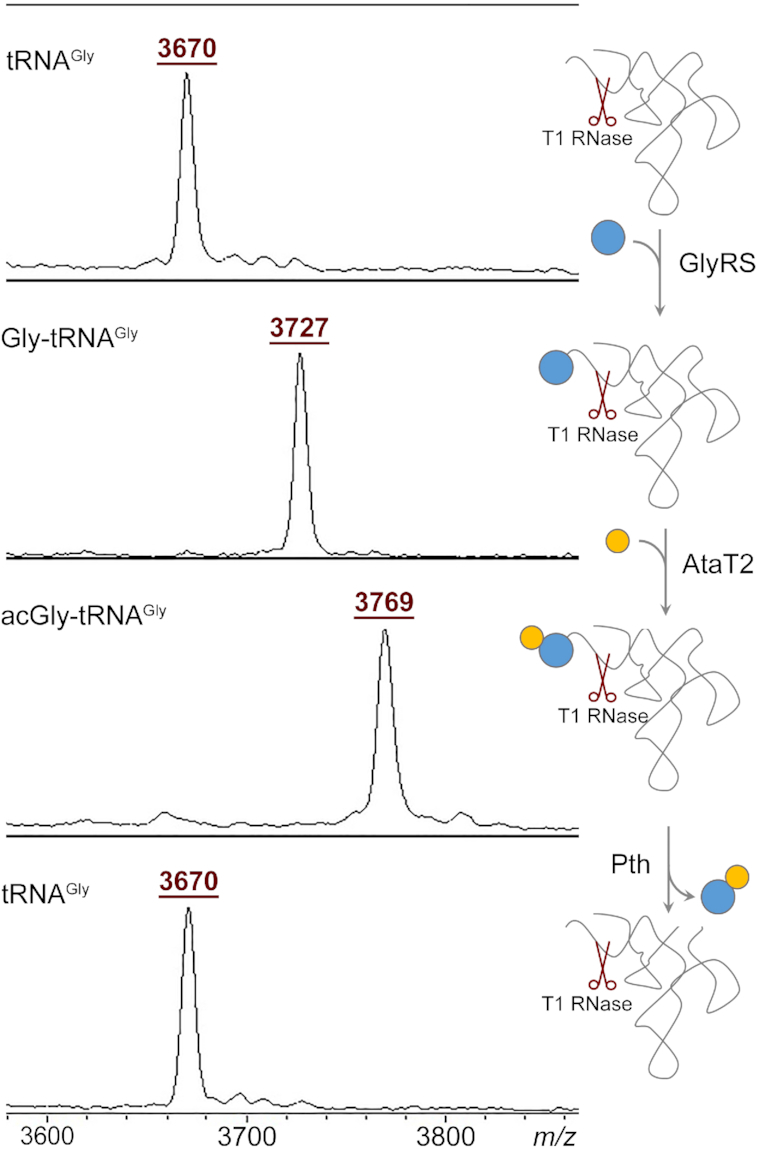
AtaT2 acetylates glycyl residue attached to tRNA^Gly^. Left panels, MALDI-TOF-MS spectra of the products of the reactions depicted schematically on the right. tRNA^Gly^ was sequentially treated with glycyl tRNA synthetase (GlyRS), AtaT2, and Pth enzyme. At each step, an aliquot was treated with T1 RNase and analyzed by MALDI-TOF-MS. The MH^+^ ions at *m/z* 3670, *m/z* 3727 and *m/z* 3769 correspond to the 3′-terminal fragments of glyV tRNA, glycylated glyV tRNA and glyV tRNA carrying acetylated glycyl moiety, respectively.

Previously studied GNAT toxins were shown to acetylate the aminoacyl moiety of the charged tRNA (13,14,21). To determine how exactly AtaT2 alters Gly-tRNA, we purified native glyV tRNA^Gly^ and analyzed the products of its *in vitro* modification by the purified AtaT2 using mass spectrometry. T1 nuclease treatment of uncharged glyV tRNA released an RNA fragment with *m/z* 3760, corresponding to 3′-terminal oligonucleotide 5′-UpUpUpCpCpCpGpCpTpCpCpA-3′ (Figure 4, top panel). The *in vitro* aminoacylation of glyV tRNA^Gly^ by purified glycyl-tRNA synthetase (GlyRS) increased the *m/z* value of this fragment by 57 Da, corresponding to the addition of glycyl moiety (Figure 4, second panel from the top). When aminoacylated glyV tRNA^Gly^ was treated with AtaT2 in the presence of acetyl-CoA, the MH^+^ ions corresponding to this fragment increased further by 42 Da, matching the addition of an acetyl group. AtaT2 did not modify the control Ile-tRNA^Ile^ (Supplementary Figure S5). N-acetylated aminoacyl-tRNA resembles peptidyl-tRNA and can serve as a substrate for peptidyl-tRNA hydrolase (Pth). The addition of Pth to AtaT2-modified Gly-tRNA resulted in the accumulation of a fragment, corresponding to uncharged tRNA^Gly^ (Figure 4, bottom panel), confirming that AtaT2 modifies Gly-tRNA by acetylating the α amino group of the glycyl moiety.

## DISCUSSION

In this work, we characterized the mechanism of toxicity of *E. coli* O157:H7 AtaT2, a member of extensive but insufficiently studied family of GNAT toxins. Since AtaT2 has only distant similarity to other GNAT toxins with known specificity, we reasoned that it might have a different target and/or mode of action. Because the *in vitro* approach for toxin target identification alone may lead to erroneous conclusions (38), we combined *in vivo* and *in vitro* approaches to identify the AtaT2 target. We showed that *in vivo* and *in vitro*, AtaT2 acts as a potent inhibitor by arresting translation, specifically at Gly codons. We further established that AtaT2 acetylates the α-amino group of the glycyl moiety of Gly-tRNA^Gly^, making it unable to participate in the translation process.

The extended dwelling time at glycine codons results in an overall decrease in the number of ribosomes that are able to complete translation (Figure 2B). Because in the Ribo-seq data, the ribosome density was not confined exclusively to Gly codons, the ribosomes apparently can translate through them in the presence of AtaT2 though with reduced efficiency. Therefore, a fraction of Gly-tRNA^Gly^ in the cell remains unmodified by the toxin at our conditions. Several reasons could account for this effect: i) the amount of the toxin could be insufficient to modify all Gly-tRNAs; ii) a salvage pathway, e.g. hydrolysis of acetyl-Gly-tRNA by Pth (Figure 4), could regenerate intact tRNAs^Gly^, making them available for new rounds of aminoacylation; and iii) misincorporation of a near-cognate aminoacyl-tRNA at hungry glycine codons or frameshifting could allow the ribosome to continue translation past the pause site.

The expression of type II TAs is negatively regulated by cognate toxin–antitoxin complexes (10). Strikingly, the AtaR2 antitoxin sequence does not contain any glycyl residues. As an expected consequence, the *in vitro* translation of AtaR2 is not inhibited in the presence of AtaT2 (Supplementary Figure S6). We speculate that continued synthesis of the antitoxin in the presence of the toxin may help to maintain a negative feedback loop that prevents the excess synthesis of AtaT2 and facilitates the recovery of cells from the toxin action.

The TA systems are ubiquitously present on plasmids and in bacterial and phage genomes (8,9). Stable maintenance of a particular TA may be indicative of its potential beneficial role to the host or result from a selfish, addictive behavior of its products. The role of chromosomally-encoded TA modules, including AtaRT2 described here, remains unclear as recent studies have questioned the previously postulated functions of TAs (6). It was proposed that chromosomally encoded AtaRT and AtaRT2 evolved as anti-addiction modules as antitoxin AtaR can counteract the toxic action of plasmid-borne AtaTpl (21). Yet, the simultaneous maintenance of an antitoxin and the potentially harmful toxin in the genome solely to counteract plasmid addiction modules seems redundant, suggesting that there may be alternative roles for chromosome-encoded TA modules. The fact that three GNAT toxins with an established mechanism of action, AtaT, ItaT, and AtaT2, recognize distinct sets of tRNAs, i.e. formyl methionine (15), isoleucine (13) and glycine (this work), respectively, suggests that among hundreds of yet uncharacterized toxins there could be those that specifically target other if not all individual aminoacyl tRNA. It is tempting to speculate that chromosome-encoded GNAT toxins targeting different aminoacyl tRNAs serve as global regulators responsible for fine-tuning of translation efficiency under stress conditions.

## DATA AVAILABILITY

Raw data from Ribo-Seq experiments are deposited at GEO under accession number GSE148424. A custom python script for Ribo-Seq data analysis is available at github.com/bikdm12/AtaT2-ribo-seq.

## Supplementary Material

gkaa551_Supplemental_File
