# Supplementary material for: Mechanism of translation inhibition by type II GNAT toxin AtaT2
Source: Nucleic Acids Res. 2020 Jun 29;48(15):8617–25. doi: 10.1093/nar/gkaa551 (PMC7470980; doi:10.1093/nar/gkaa551)
Supplement: gkaa551_Supplemental_File [file gkaa551_supplemental_file.docx]

Supplementary materials

**Mechanism of translation inhibition by type II GNAT toxin AtaT2**

Stepan V. Ovchinnikov, Dmitry Bikmetov, Alexei Livenskyi, Marina Serebryakova, Brendan Wilcox, Kyle Mangano, Dmitrii I. Shiriaev, Ilya A. Osterman, Petr V. Sergiev, Sergei Borukhov, Nora Vazquez-Laslop, Alexander S Mankin, Konstantin Severinov, Svetlana Dubiley


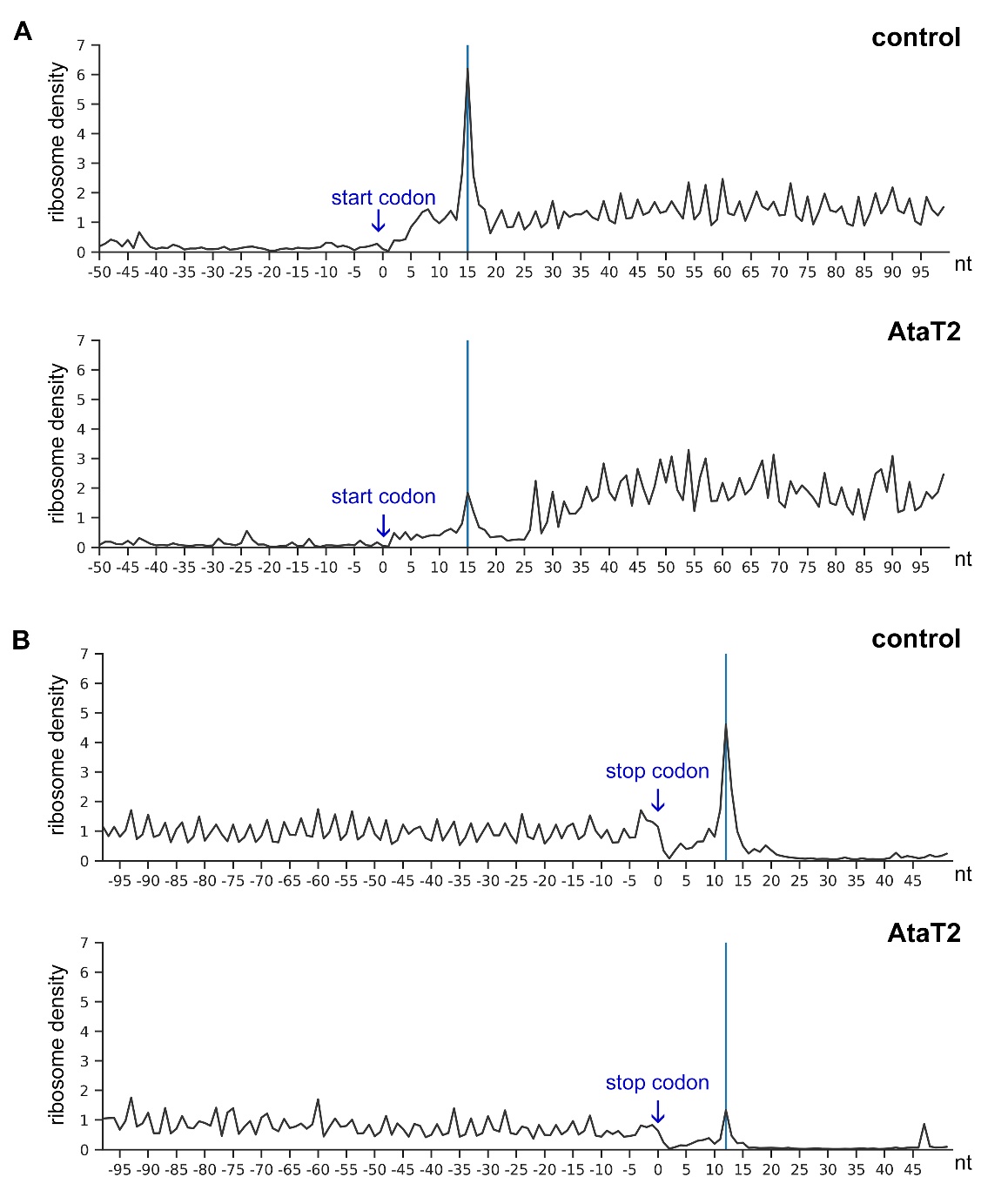


**Figure S1. Ribosome density plots averaged for all ORFs and aligned at the start (A) or the stop (B) codons (metagene analysis).** Zero-point on the horizontal axis corresponds to the first nucleotide of the start codon (A) or the stop codon (B), respectively. In (A), the first major peak (at 15 nt, marked by the blue vertical line) corresponds to the 3’-ends of footprints with the start codon located in the ribosomal P site. The last major peak in (B) (12 nt, marked by the blue vertical line) corresponds to the 3’-ends of footprints with the stop codon located in the ribosomal A site.


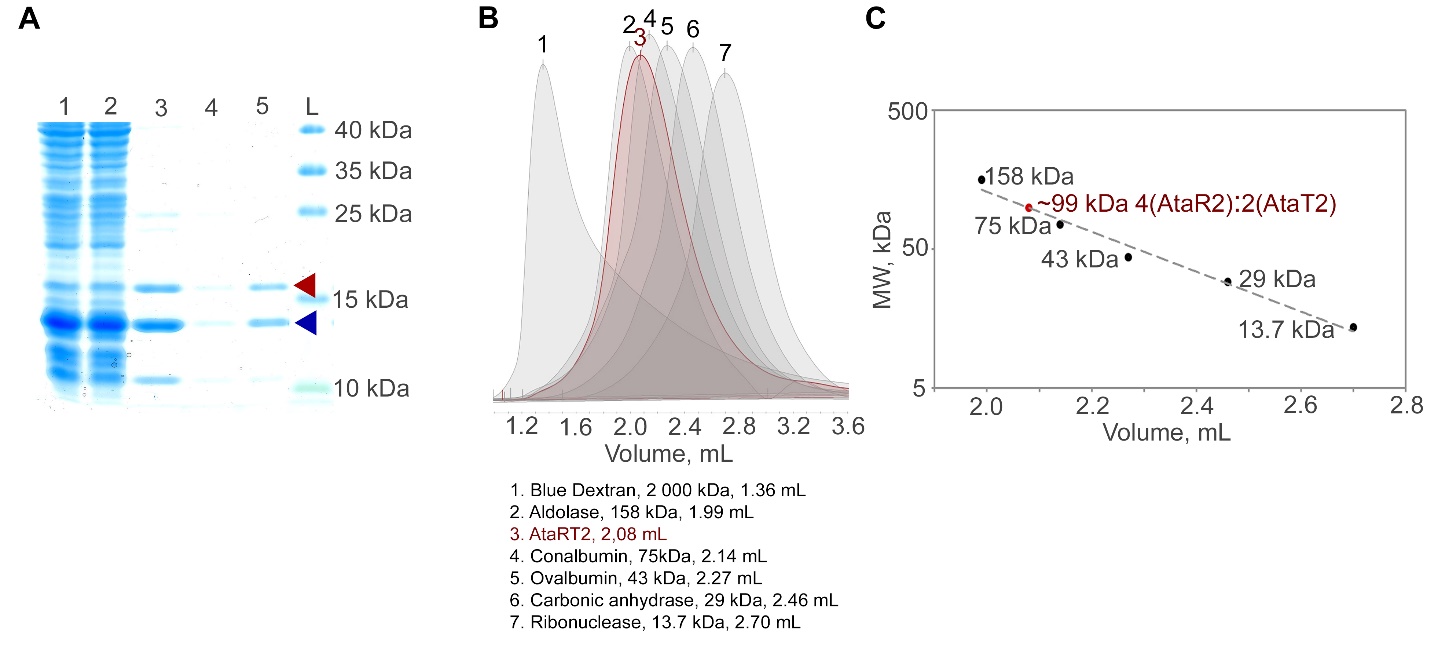


**Figure S2. AtaT2 forms a complex with AtaR2.**

(A) Antitoxin AtaR2 N-terminally tagged with Strep-Tag, and AtaT2 toxin C-terminally tagged with His6 were co-expressed and subjected to tandem affinity chromatography purification. Protein samples were separated by 12% SDS-PAGE and stained by Coomassie. Lane L, Protein ladder; lane 1, clarified lysate of induced BL21(DE3) cells carrying pET-str-*ataRT2*-his plasmid; lane 2, Co^2+^-TALON flow-through fraction; lane 3, Co^2+^-TALON wash; lane 4, Co^2+^-TALON eluate; lane 5, an unbound fraction of Co^2+^-TALON eluted protein after incubation with Strep-Tactin agarose; lane 6 – Strep-Tactin wash; lane 7, Strep-Tactin eluate. Red and blue arrows indicate positions of AtaT2 toxin and AtaR2 antitoxin on the gel, respectively; the identity of both proteins was confirmed by mass-spectrometry sequencing.

(B) The results of size-exclusion chromatography of the AtaRT2 complex and the molecular weight standards on the Superdex 200-10/300GL column. FPLC traces of the protein molecular weight standards are shown in grey, of the AtaRT2 - in red.

(C) The calculated apparent molecular weight of the AtaR2-AtaT2 complex.


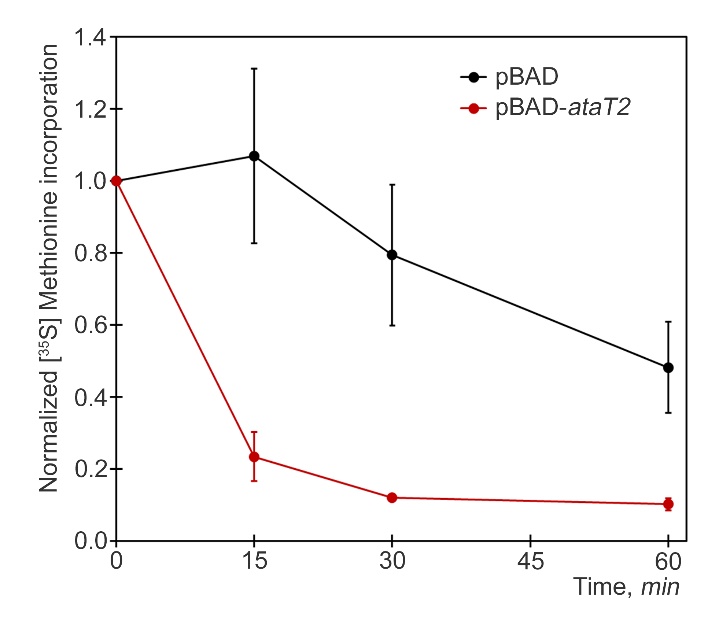


**Figure S3. Metabolic labeling of *E. coli* BW25113 cells expressing AtaT2 toxin.**

Cells harboring pBAD-*ataT2* or empty vector control were induced with arabinose. At the indicated times, aliquots of the cultures were combined with [^35^S]-L-Met, incubated for 1 min, and incorporation of the radioactivity into acid-insoluble fraction was measured. The graph shows the mean value of incorporated radioactivity normalized to the empty vector control prior to the induction (time point 0). Standard errors of the mean calculated from two independent experiments are indicated.


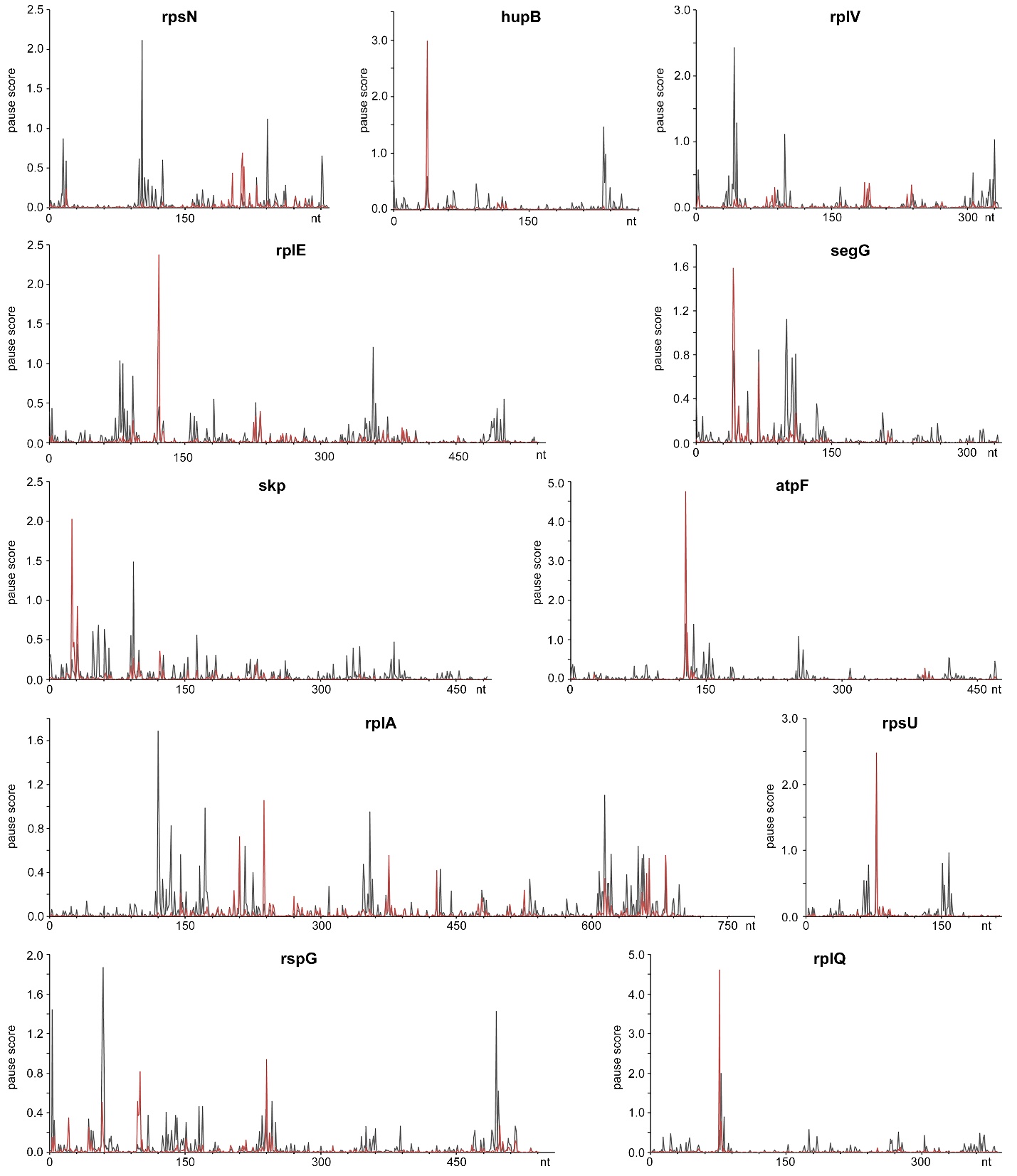


**Figure S4. Pause score profiles for individual genes in selected representative ORFs.**

Ribosomal pause score profiles observed in cells expressing AtaT2 (red) and in control (dark grey). The pause score value was calculated as ribosome density at each position of the gene divided by average ribosome density on this gene.


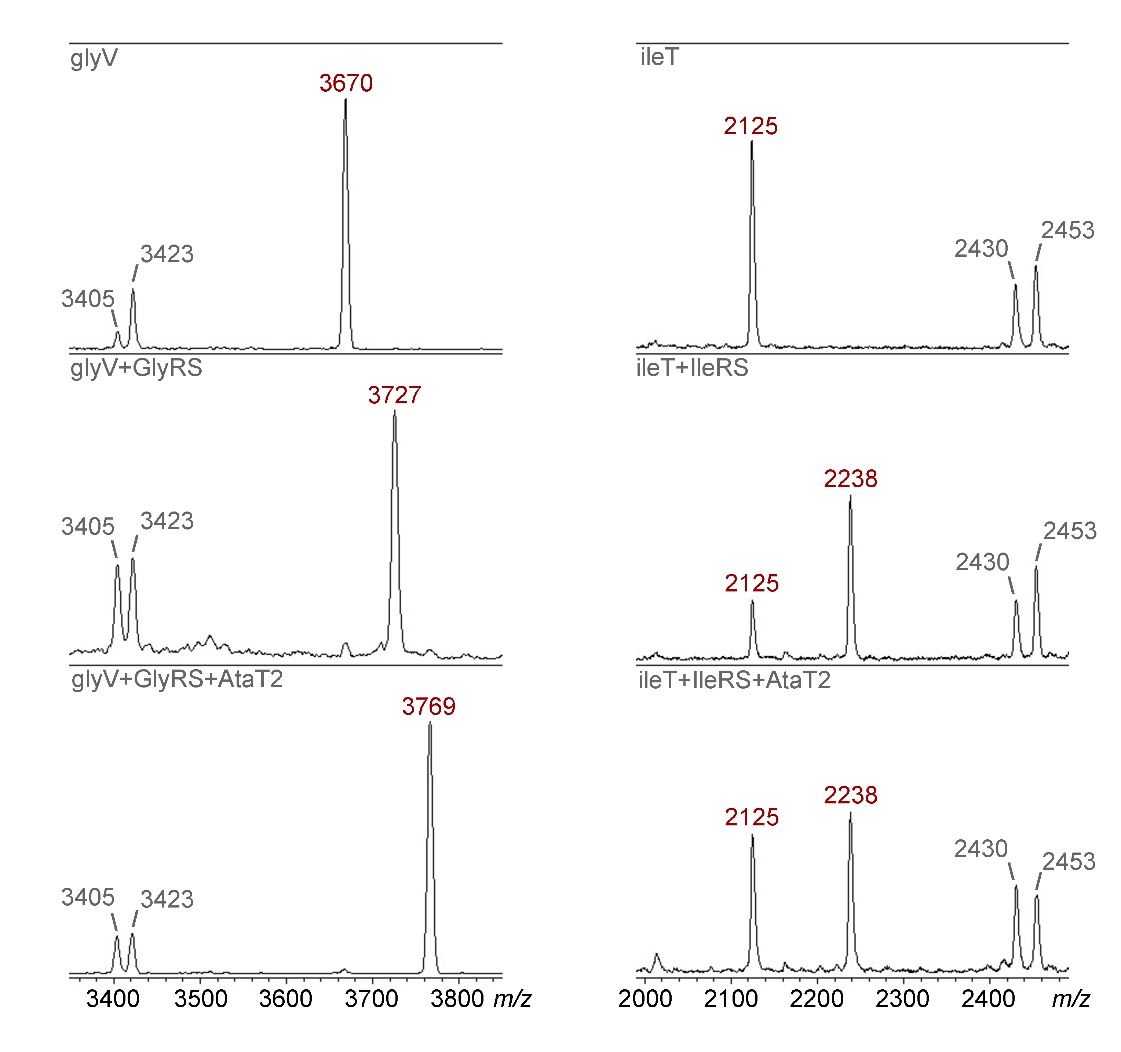


**Figure S5. In vitro modification of charged tRNAs with AtaT2.**

tRNA^Gly^ (glyV, left panels) and tRNA^Ile^ (ileT, right panels) were incubated with cognate amino acids, ATP and tRNA synthetases in the presence or absence of acetyl-CoA and AtaT2. Modified tRNAs were treated with the T1 endoribonuclease and analyzed with MALDI-TOF-MS. The peaks marked with red-coloured font correspond to 3’-terminal fragments of tRNAs. The peaks labeled with grey-coloured font correspond to internal fragments of tRNAs. Peak shifts of 57 and 113 Da match the addition of glycine and isoleucine, respectively. A 42 Da peak shift corresponds to the addition of an acetyl group.


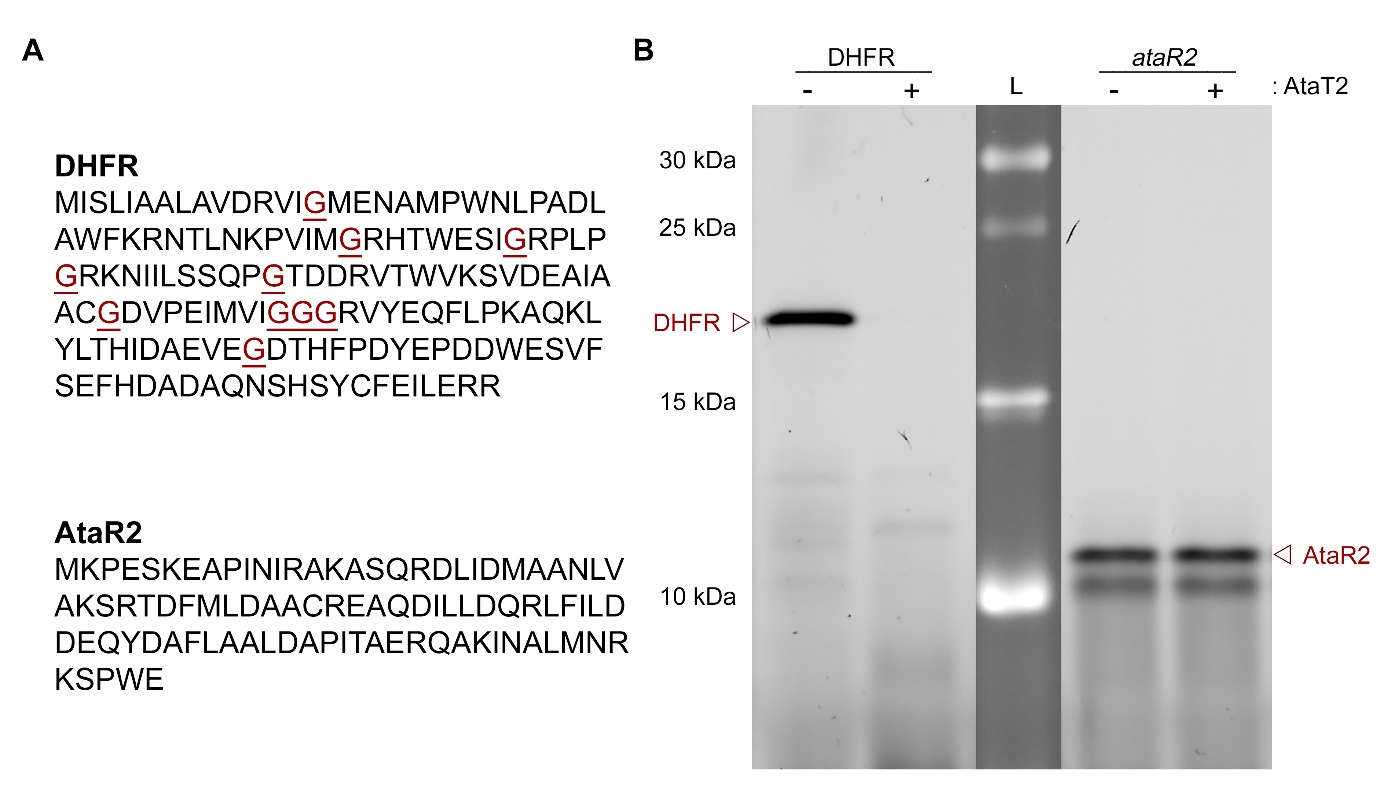


**Figure S6. *In vitro* transcription-translation reactions using *ataR2* and DHFR templates in the presence or absence of AtaT2 toxin.**

(A) Amino acid sequences of DHFR and AtaT2. Glycine residues are indicated in red.

(B) *In vitro* translation assay of the *ataR2* and DHFR mRNAs. Reactions were supplemented with acetyl-CoA and FAM-labeled lysine and carried out in the presence or absence of AtaT2, as indicated above the lanes. Reaction products were separated by 12% SDS-PAGE and visualized by fluorescent imaging. Lane L, Cy5-labeled standard protein ladder. The position of bands, corresponding to the full-length AtaR2 and DHFR are indicated by arrowheads.

**Supplemetary Table S1. Primers used in the study**

| Name | Sequence, 5’-3’ |
| --- | --- |
| AtaT2-F-EcoRI | TTATGAATTCAGGAGGAATTAAAAATGGGAATAACGGCTCCTACT |
| AtaR2-F-EcoRI | TTATGAATTCAGGAGGAATTAAAAATGAAACCGGAAAGCAAAGAAGCT |
| AtaT2-R-HindIII | TTATAAGCTTTTACTCCAGAGTAATGGGATACAGTAAGGT |
| AtaT2-Y139A-F | GAATGAAAAAGCCCATACGTTTGCTAAATCGCTGGGCTTTATCC |
| AtaT2-Y139A-R | GGATAAAGCCCAGCGATTTAGCAAACGTATGGGCTTTTTCATTC |
| AtaR2-F-BamHI | TTATAGGATCCGATGAAACCGGAAAGCAAAGAA |
| AtaT2-R-XhoI | TTATACTCGAGTTACTCCAGAGTAATGGGATACAGTAA |
| T7-ataR2-F | AATTAATACGACTCACTATAGGGTCTAGAAATAATTTTGTTTAACTTTAAGAAGGAGATATACATATGAAACCGGAAAGCAAAGAAG |
| T7-ataR2-R | CGTTAGGGGAGTAGGAGC |
| GlyRS_F-Bam | TTATAGGATCCGATGCAAAAGTTTGATACCAG |
| GlyRS_R-Xho | TTATACTCGAGTTGCAACAGCGAAATATCCGC |
